# Prevalence and etiological agents for chronic suppurative otitis media in a tertiary hospital in Tanzania

**DOI:** 10.1186/s13104-019-4483-x

**Published:** 2019-07-17

**Authors:** Zephania Saitabau Abraham, Daudi Ntunaguzi, Aveline Aloyce Kahinga, Kassim Babu Mapondella, Enica Richard Massawe, Emmanuel James Nkuwi, Aslam Nkya

**Affiliations:** 1 Department of Surgery, University of Dodoma, College of Health and Allied Sciences, Box 259, Dodoma, Tanzania; 2 Department of Otorhinolaryngology, Muhimbili University of Health and Allied Sciences, Box 65001, Dar es Salaam, Tanzania; 3 Department of Microbiology and Immunology, University of Dodoma, College of Health and Allied Sciences, Box 259, Dodoma, Tanzania

**Keywords:** Prevalence, Aetiology, Chronic suppurative otitis media, Muhimbili, Tanzania

## Abstract

**Objective:**

Chronic suppurative otitis media is among the most common otological condition reported in otorhinolaryngology practice commonly attributing to preventable hearing loss. The aim of this study was to determine the prevalence and etiological agents for chronic suppurative otitis media in our department.

**Results:**

A total of 5591 patients were recruited in this study and only 79 (1.4%) had chronic suppurative otitis media. A male preponderance 43 (54.4%) was noted in this study and the left ear (58.2%) was more commonly affected compared to the right ear. Central perforation was the commonest pattern of presentation and was reported in 53% of cases though none had attic perforation. Of the 81 processed ear swabs, microbial growth was seen in majority 80 (98.8%) whilst one sample showed no microbial growth whereas 52.5% had polymicrobial growth. Among the isolates, most were gram negative species accounting for 59.7% while gram positive bacteria accounted for 25.6% and fungi accounted for 14.7%. Most of these isolates were facultative anaerobes. *Klebsiella pneumoniae* (20.2%) was the commonest isolates while *Escherichia coli* and *Pseudomonas aeruginosa* were equally least isolated (10.9%). Tested isolates were most sensitive to Ciprofloxacin, Gentamycin, Ceftriaxone and Amikacin and least sensitive to Amoxicillin/clavulanic acid and Ampicillin.

**Electronic supplementary material:**

The online version of this article (10.1186/s13104-019-4483-x) contains supplementary material, which is available to authorized users.

## Introduction

Chronic suppurative otitis media (CSOM) is a disease condition associated with chronic inflammation of the middle ear cleft characterized by persistent perforation of the tympanic membrane with recurrent or persistent mucopurulent otorrhoea. The duration of otorrhoea for classifying CSOM has been the subject of discussion among Otorhinolaryngologists with duration ranging from between 3 weeks to 3 months. In this study the period taken will be at least 2 weeks in accordance with World Health Organization recommendation [1].

It is also one of the leading causes of preventable hearing loss worldwide and in most cases it is a sequela of improperly attended acute otitis media and it consequently impairs one’s quality of life [1, 2].

Hearing loss is common among patients with CSOM and exceeds 30 dB and with a tendency to occur in about 50 to 60% of such patients [3, 4]. Conductive hearing loss is typically moderate to severe in up to two-thirds of patients and being marked at low frequencies and with increased bone conduction threshold tendency [4–12].

The site of the perforation corresponds to degree of hearing loss, with posterior perforations having greater decibel level loss probably as a result of loss of protection of the round window membrane from impinging sound pressure waves [7].

Methicillin resistant *Staphylococcus aureus* (MRSA) and methicillin-susceptible *Staphylococcus aureus* (MSSA) have been found in pus swab cultures of patients with CSOM and this poses a significant challenge in its medical management due to its resistance to commonly used antibiotics [13, 14].

On the other hand, there has been increased incidence of multi drug resistance which is another hindrance as they are related to increased complications associated with CSOM [13, 14]. In Tanzania, most of the available studies were based on the prevalence of this condition and very few studies have been conducted to look on the bacteriological and sensitivity pattern to antibiotics which is pertinent in the management of such patients.

## Main text

### Methods

#### Study design, participants and sampling method

This was a hospital based descriptive cross-sectional study conducted between September 2015 and February 2016 and included 1200 patients who attended Otorhinolaryngology (ORL) Department at Muhimbili National Hospital. Convenient sampling technique was utilized.

#### Inclusion and exclusion criteria

All adult patients who consented to participate in the study and those under the age of 18 years whose parents/caretakers consented on their behalf. Patients on regular follow up were excluded.

#### Specimen collection procedures

Pus swab was collected from the external auditory canal and introduced into Amies transport medium bottle and sent for laboratory analysis.

#### Laboratory procedures

From each specimen, a portion was subjected to primary gram stain for pus cells and possible organism while the remaining portions were inoculated into Blood agar (Oxoid, UK), and MacConkey agar (Oxoid, UK) and incubated aerobically at 37 °C for 24–48 h.

#### Identification of bacterial pathogens

Identification of pathogens was based on Microscopy (Gram stain, shape, cells arrangement) and colony characteristics (colony morphology, hemolysis on blood agar, changes in the physical appearance of the differential media). Organisms from discrete colonies were cultured into Nutrient Agar (Oxoid, UK) for subsequent. Biochemical tests. Gram positive isolates were tested for catalase and Coagulase tests while biochemical tests for gram negative isolated bacteria were tested for oxidase, Triple sugar Iron (TSI), Sulphur indole and motility (SIM), urease production and citrate utilization [15].

#### Antimicrobial susceptibility testing

Antibiotic susceptibility pattern of isolated bacteria pathogens was performed using modified Kirby Bauer disc diffusion method according to the guidelines of the clinical and Laboratory Standard Institute (CLSI) [16].

A colony suspension with concentration equivalent to 0.5 McFarland solution was prepared for each identified isolate and inoculated into Mueller–Hinton-Agar (Oxoid, UK). Appropriate Selected Antibiotic discs were placed onto the media and incubated at 37 °C for 24 h.

Gram positive isolates were tested against Ampicillin (10 µg), Amoxicillin/clavulanate (20/10 µg), Ceftriaxone (30 µg), Gentamycin (10 µg), Ciprofloxacin (5 µg), Trimethoprim/sulfamethoxazole (1.25/23.75 µg), Chloramphenicol (30 µg), Amikacin (17 µg) and Cephalexin (18 µg).

Gram negative organisms were tested against Ampicillin (10 µg), Amoxicillin/clavulanate (20/10 µg), Ceftriaxone (30 µg), Gentamicin (10 µg), Ciprofloxacin (5 µg), Triomethoprim/sulfamethoxazole (1.25/23.75 µg) and Chloramphenicol (30 µg). Reference stains used for quality control were *Staphylococcus aureus* (American Type Culture Collection; ATCC 25922 and ATCC 29213), *Escherichia coli* (ATCC 25922), *Pseudomonas aeruginosa* (ATCC2785) [15, 16].

#### Data analysis

Data analysis was done using the Statistical Package for Social Sciences version 21. P-value of < 0.05 was considered statistically significant.

### Results

#### Demographic characteristics of study participants

A total of 5591 patients were recruited and include both in and out patients. The age distribution ranged from 7 months to 82 years. The mean age (Standard Deviation; SD) of study participants was 12.9 (SD ± 7.9). Majority of patients were over 40 years old (28.1%) and the minority (4.9%) were between 16 and 20 years of age. Slight female predominance (52.4%) was noted in this study (Table 1).

**Table 1:** Demographic characteristics of the eligible patients

Variable	Frequency	Percentage
Age group (years)		
< 5	1468	26.3
6–10	416	7.4
11–15	332	5.9
16–20	274	4.9
21–25	445	8.0
25–30	391	7.0
31–35	348	6.8
36–40	342	6.1
> 40	1575	28.1
Sex		
Male	2663	47.6
Female	2958	52.4
Total	5591	100

#### Prevalence of chronic suppurative otitis media

Table 2 shows the prevalence of CSOM with regard to age and gender. Of the 5591 patients, 79 (1.4%) had CSOM. The proportion of CSOM was highest in patients aged between 11 and 15 years (3%) and least in those aged above 40 years (0.6%). A male predominance of 54.4% was also noted.

**Table 2:** Prevalence of chronic suppurative otitis media by age and sex

Variables	Frequency	CSOM	Proportion (%)
Age group (years)			
< 5	1468	21	1.4
6–10	416	9	2.2
11–15	332	10	3.0
16–20	274	7	2.6
21–25	445	8	1.8
26–30	391	4	1.0
31–35	348	8	2.3
36–40	342	3	0.9
> 40	1575	9	0.6
Sex			
Male	2663	43	1.6
Female	2958	36	1.2
Total	5591	79	1.4

#### Lateralization and types of tympanic membrane perforation

Unilateral involvement (97.5%) was commoner than bilaterality (2.5%). Left ear infection and bilaterality accounted for 58.3% and 2.5% of cases respectively. Central perforation (53.2%) was the predominant type.

#### Distribution of bacterial and fungal isolates

**Fig. 1:**
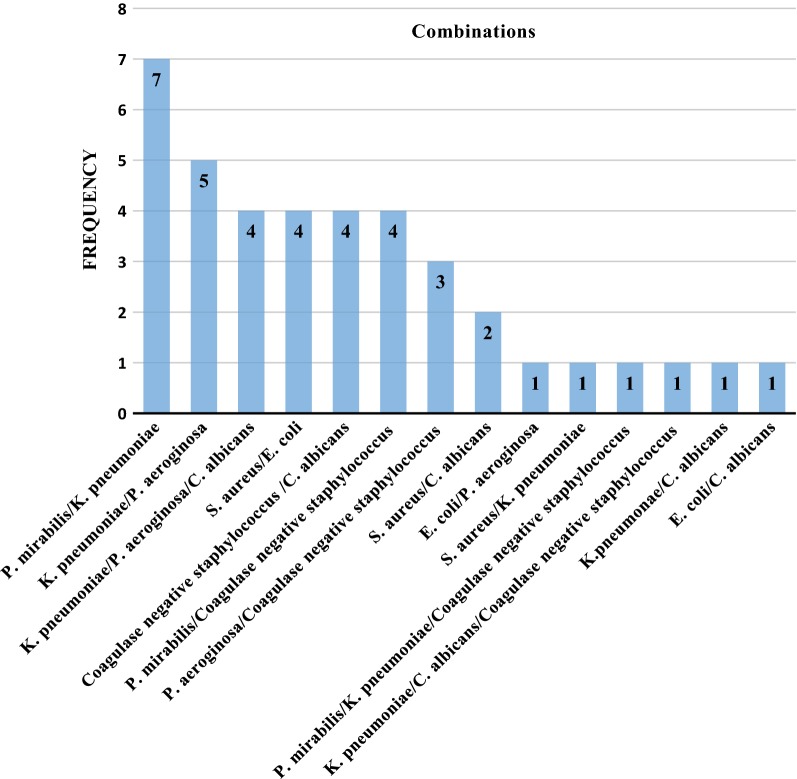
Polymicrobial combination of isolates

Figure 1 depicts distribution of bacterial and fungal isolates. Of the 79 patients with CSOM in whom a pus specimen was collected for culture, 98.8% yielded a positive culture. Most of culture growth yielded polymicrobial growth (52.5%), in which the most common pathogen was a mixture of *Proteus mirabilis* and *Klebsiella pneumoniae* (16.7%) (Fig. 1). As for single microbial growth, *Escherichia coli* and *Staphylococcus aureus* were equally prevalent (21.1%).

Overall, Gram negative bacteria were more common (59.7%) and least was fungi (*Candida albicans*) (14.7%) (Additional file 1: Figure S1). Among Gram-negative bacteria, *Klebsiella pneumoniae* was the most prevalent (33.8%), Among Gram-positive bacteria, Coagulase negative *Staphylococcus* spp. was the commonest (54.5%). Generally, of all isolates, *Klebsiella pneumoniae* was the commonest (20.2%) followed by *Proteus mirabilis* (17.8%) (Additional file 1: Figure S1).

#### Susceptibility pattern of bacterial isolates

*Klebsiella pneumoniae* was highly susceptible to Gentamicin (80.8%) and moderately susceptible to Ceftriaxone (73.1%) and Ciprofloxacin (61.5%), whereas none among these two isolates were susceptible to Ampicillin and Amoxicillin/clavulanic acid. (Additional file 1: Table S1).

### Discussion

CSOM is one of the most important public health concerns particularly in developing countries [1]. Early identification and proper management of these cases is of vital importance particularly in alleviating complications associated with this disease. In the present study, the proportion of patients with chronic suppurative otitis media was found to be 1.4%. This observation was slightly lower than the previous hospital based study in Tanzania [17] and other community based studies conducted in Dar es Salaam and Northern Tanzania [18, 19]. Elsewhere hospital based studies in Sudan [20] and community based studies in India [21] and Solomon Islands [22] had reported higher prevalence than what has been found in our study. This could be attributed to differences in sample size and sampling techniques. Similar to other studies [1], subjects aged below 5 years of age in this study had high proportion of the disease accounting for 26.6% of all cases of chronic suppurative otitis media. This may be explained by their relative immature immunity, leading to recurrent upper respiratory tract infections and their relatively short and horizontal eustachian tube making them prone to infection. Another key similarity to other studies is that, this study found males to be more affected by CSOM compared to females with male to female ratio of 1.2:1 and this was statistically significant. Such finding shows resemblance to other studies done elsewhere [22] though differing with observed findings done elsewhere with female propensity [23] and equal gender predominance [10]. Since this study employed random selection of subjects, a male predominance might be an incidental findings and still no known anatomical and genetic differences between male and females pertaining the ear exists.

This study found left ear disease to account for the majority of the cases (58.2%) with bilateral involvement accounting for the least (2.5%) number of cases which is similar to what was found by Olowookere et al. [23] though differing with findings established by Taipale et al. and an Aboriginal study [3, 24] with bilateral predominance. Predominance of the left ear may be due to random selection of the study cases but no genetic or structural differences have been identified between the right and left ear.

This study found central perforation (tubotympanic type) to be predominant (53%) while none of patients had attic perforation. Such findings correlate closely with what was found elsewhere [22, 25].

An analysis of 81 ear pus swabs for culture revealed microbial growth in 98.8% while the rest had no growth, mixed infections (polymicrobial) accounted for majority of the cases. The commonest mixed infections were of *Proteus mirabilis* and *Klebsiella pneumoniae*, followed by *Klebsiella pneumoniae* and *Pseudomonas aeruginosa*. Predominance of mixed bacterial infection is in line to previous finding by Gupta et al. [26] from India which found that about 70% of the CSOM had polymicrobial infections. The polymicrobial nature of the disease may well be explained by the fact that the perforated ear drum makes easier for coliform bacteria such as *Escherichia coli* and *Pseudomonas aeruginosa* which are associated with wet and poor hygienic environments and fungi from the external ear to migrate into the middle ear and proliferate.

This polymicrobial nature of the disease with both gram positives, gram negative and fungi necessitates the need for cover with antibacterial drugs with action against both gram positive and gram negative with an addition of antifungal drug.

This study also observed that gram negative bacteria accounted for the majority of the isolates while fungi accounted for the least. Most of the isolates were facultative anaerobes similar to what has been reported in other studies [17, 27] with other researchers observing gram positive *Staphylococcus aureus* as the predominant isolates [28, 29].

Isolation of coliform bacteria *Escherichia coli* and *Klebsiella pneumoniae* which are known to be fecal bacteria and *Pseudomonas aeruginosa* which is associated with wet environmental conditions suggests people are at high‑risk of infection due to poor hygienic environment. These findings were in line with observational study done in Nigeria by Bakari et al. [27] which had *Klebsiella pneumoniae* as the commonest isolated bacteria. But most authors elsewhere have reported *Pseudomonas aeruginosa* as the commonest isolates [17, 20, 26, 30, 31] while Ferede et al. [32] in their study found *Proteus species* followed by *Staphylococcus aureus* as their commonest isolates.

Antimicrobial susceptibility test was carried out for all the aerobic isolates (except for *Coagulase negative staphylococcus*). Ciprofloxacin was found to be the most effective drug as it has been reported in other studies [27, 33]. This study has thus elucidated the prevalence and etiological profile for CSOM at MNH which is the largest country’s tertiary hospital.

### Conclusions

The prevalence of CSOM at MNH appears to be in line with what has been reported in other parts of the world. A male predominance was found and the left ear was more commonly affected than the right ear. Prevalent polymicrobial nature and antimicrobial resistance among isolates in CSOM cases warrants the need for culture and sensitivity of pus isolates. Ciprofloxacin, Gentamicin, Ceftriaxone and Amikacin are highly recommended as the first line management in patients with CSOM, with consideration of appropriate antifungals for possible fungal etiology as per our study findings.

## Limitations

Due to lack of anaerobic culture facility we couldn’t further explore the role of anaerobic bacteria in chronic suppurative otitis media. Contaminants were also identified and this is explained by the nature of swab which was employed.

## Additional file

**Additional file 1: Figure S1.** Distribution of microbial isolates. **Table S1.** Susceptibility pattern of bacteria isolated from CSOM.

## Data Availability

All relevant data pertinent to this research can be obtained from the corresponding author upon a reasonable request.

## Abbreviations

ATCC: American Type Culture Collection
CLSI: Clinical and Laboratory Standards Institute
CSOM: chronic suppurative otitis media
MNH: Muhimbili National Hospital
MRSA: methicillin-resistant *Staphylococcus aureus*
MSSA: methicillin-susceptible *Staphylococcus aureus*
ORL: otorhinolaryngology
SD: standard deviation
SIM: sulphur indole and motility
TSI: triple sugar iron
UK: United Kingdom
