# Supplementary material for: Prevalence and etiological agents for chronic suppurative otitis media in a tertiary hospital in Tanzania
Source: BMC Res Notes. 2019 Jul 17;12:429. doi: 10.1186/s13104-019-4483-x (PMC6637475; doi:10.1186/s13104-019-4483-x)
Supplement: Supplementary file 1 — Additional file 1: Figure S1. Distribution of microbial isolates. Table S1. Susceptibility pattern of bacteria isolated from CSOM. [file 13104_2019_4483_MOESM1_ESM.docx]

**Additional Data:**

**Figure S1: Distribution of Microbial Isolates**


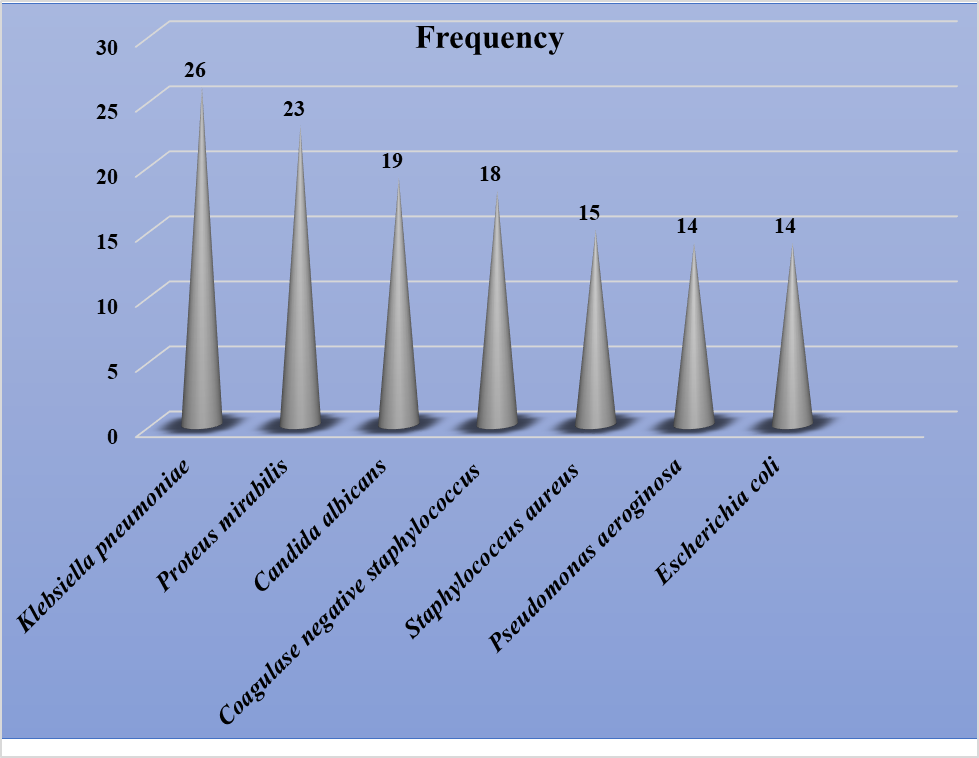


The figure S1 above shows that of the 79 ear pus swabs; Gram-negative bacteria, *Klebsiella pneumoniae* was the most prevalent (33.8%), followed by *Proteus mirabilis* (29.9%), *Pseudomonas aeruginosa* and *Escherichia coli* (18.2%). Among Gram-positive bacteria, Coagulase negative *staphylococcus spp* was the commonest (54.5%), followed by *Staphylococcus aureus* (45.6%). Generally, of all isolates, *Klebsiella pneumoniae* was the commonest (20.2%) followed by *Proteus mirabilis* (17.8%)

**Table S1: Susceptibility pattern of bacteria isolated from CSOM**

| Antibiotics | % of bacteria susceptible | | | | |
| --- | --- | --- | --- | --- | --- |
|  | *K.pneumoniae* | *P.aeruginosa* | *E.coli* | *S.aureus* | *P.mirabilis* |
| AMP | 0 | 0 | 0 | 0 | 4.4 |
| CN | 80.8 | 100 | 50 | 46.7 | 65.2 |
| AMC | 0 | 0 | 14.3 | 13.3 | 4.4 |
| CHL | 42.3 | 21.4 | 71.4 | 53.3 | 39.1 |
| CIP | 61.5 | 100 | 78.6 | 73.3 | 78.3 |
| STX | 26.9 | 50 | 28.6 | 26.7 | 26.1 |
| CRO | 73.1 | 57.1 | 71.4 | 40 | 73.9 |
| AMK | 69.2 | 78.6 | 64.3 | 26.7 | 60.9 |
| CEP | 46.2 | 0 | 35.7 | 60 | 60.9 |

AMP= Ampicillin, CN=Gentamicin, AMC=Amoxicillin-clavulanic acid, CHL =Chloramphenicol, TET=Tetracycline, CIP=Ciprofloxacin, STX=Trimethoprim-sulfonamide, CRO=Ceftriaxone, AMK=Amikacin, CEP= Cephalexin

The table S1 above shows that, *Klebsiella pneumoniae* was highly susceptible to Gentamicin (80.8%) and moderately susceptible to Ceftriaxone (73.1%) and Ciprofloxacin (61.5%) whereas none were non susceptible to Ampicillin and Amoxicillin/clavulanic acid. *Pseudomonas aeruginosa* was highly susceptible to Gentamicin and Ciprofloxacin (100%) while they were all non-susceptible to Ampicillin, Amoxicillin/clavulanic acid, and Cephalexin. *Escherichia coli* was highly susceptible to Ciprofloxacin (78.6%), followed by Chloramphenicol and Ceftriaxone each accounting for 71.4% of cases; and all were non susceptible to Ampicillin. *Staphylococcus aureus* was highly susceptible to ciprofloxacin (73.3%) while none was susceptible to ampicillin. *Proteus mirabilis* was highly susceptible to Ciprofloxacin (78.3%) but moderately to Ceftriaxone (73.9%), All had poor susceptibility to Ampicillin and Amoxicillin clavulanic acid.
